# Disparities in triage and management of the homeless and the elderly trauma patient

**DOI:** 10.1186/s40621-020-00262-1

**Published:** 2020-07-13

**Authors:** Kathryn B. Schaffer, Jiayan Wang, Fady S. Nasrallah, Dunya Bayat, Tala Dandan, Anthony Ferkich, Walter L. Biffl

**Affiliations:** Trauma Service, Scripps Memorial Hospital La Jolla, 9888 Genesee Ave., LJ601, La Jolla, CA 92037 USA

**Keywords:** Homeless, Geriatric, Trauma, Triage, Trauma activation, Emergency department

## Abstract

**Background:**

Trauma systems are designed to provide specialized treatment for the most severely injured. As populations change, it is imperative for trauma centers to remain dynamic to provide the best care to all members of the community.

**Methods:**

A retrospective review of all trauma patients treated at one Level II trauma center in Southern CA over 5 years. Three cohorts of patients were studied: geriatric (> 65 years), the homeless, and all other trauma patients. Triage, hospitalization, and outcomes were collected and analyzed.

**Results:**

Of 8431 patients treated, 30% were geriatric, 3% homeless and 67% comprised all other patients. Trauma activation criteria was met for 84% of all other trauma patients, yet only 61% of homeless and geriatric patients combined. Injury mechanism for homeless included falls (38%), pedestrian/bicycle related (27%) and assaults (24%), often while under the influence of alcohol and drugs. Average length of hospital stay (LOS) was greater for homeless and geriatric patients and frequently attributed to discharge planning challenges. Both the homeless and geriatric groups demonstrated increased complications, comorbidities, and death rates.

**Conclusions:**

Homeless trauma patients reflect similar challenges in care as with the elderly, requiring additional resources and more complex case management. It is prudent to identify and understand the issues surrounding patients transported to our trauma center requiring a higher level of care yet are under-triaged upon arrival to the Emergency Department. Although a monthly review is done for all under-triaged patients, and geriatric patients are acknowledged to be a cohort continually having delays, the homeless cohort continues to be under-triaged. The admitted homeless trauma patient has similar complex case management issues as the elderly related to pre-existing health issues and challenges with discharge planning, both which can add to longer lengths of hospital stay as compared to other trauma patients. Given the lack of social support that is endemic to both populations, these cohorts represent a unique challenge to trauma centers. Further research into specialized care is required to determine best practices to address disparities evident in the homeless and elderly, and to promote health equity in marginalized populations.

## Background

The United States Department of Housing and Urban Development annual homeless survey estimated that 553 thousand people were homeless across the nation on a given night in January 2018. California had the largest homeless population of 129,972 people and the highest prevalence of homelessness (The U.S. Department of Housing and Urban Development n.d.). San Diego County had the fourth largest homeless population in the United States, with approximately 8500 homeless people of all ages. Homelessness is often associated with many health concerns and comorbidities. Forty-three percent of the unsheltered homeless population surveyed in 2018 in San Diego reported a physical disability, 14% reported instances of substance abuse, and 43% reported instances of mental health issues (Regional Task Force on the Homeless n.d.). One study found that 80.6% of homeless admissions in New York public hospitals had a primary or secondary diagnosis of substance abuse or mental illness (Salit et al. 1998). These potentially preventable or treatable comorbidities can create a higher risk for injury and often lead to higher rates of admission, longer than average hospitalization, and are associated with significantly higher costs (Salit et al. 1998; Kushel et al. 2002).

A systematic review and meta-analysis found that more than half of homeless and marginally housed individuals had a lifetime history of traumatic brain injury (TBI), which was associated with increased suicidal ideation and risk, poorer physical and mental health, and increased health service and criminal justice system involvement. Homeless characteristics such as residential instability or substance use were associated with sustaining TBI (Stubbs et al. 2020). Another study found those homeless with a history of head injury with loss of consciousness was associated with higher odds of depression, manic or hypomanic episodes, post-traumatic stress disorder, panic disorder, mood disorder, and alcohol and drug misuse disorders (Topolovec-Vranic et al. 2017). This bidirectional relationship between TBI and homelessness is important to understand for physicians and care providers to address the impact of TBI on the homeless population.

The unhoused population faces many additional barriers to care, such as lack of health insurance, lack of access to primary and preventative care, inability or unwillingness to follow through with therapies and treatments, and only seeking healthcare in emergent situations (Hwang 2001). Aside from injury and illness, hunger, safety concerns and lack of shelter are also motivating factors for Emergency Department (ED) visits among the homeless population. Homeless adults are four times more likely to use the ED and tend to also spend more time there per visit (Salhi et al. 2017; Pearson et al. 2007; Zlotnick et al. 2013). Within the healthcare system, homeless patients have reported stigmatization, social triaging (not receiving the appropriate level of care due to societal preconceptions), disrespect, and a feeling of being invisible to providers (Martins 2008). These factors, as well as homeless persons’ vulnerability to higher rates of victimization, increase the risk of traumatic injuries requiring hospitalization (Kushel et al. 2002). Forty-three to 53 % of homeless people reported sustaining a traumatic brain injury (TBI), which is associated with several adverse outcomes including suicidal tendencies and substance abuse (Mackelprang et al. 2014; Hwang et al. 2008). After treatment, homeless individuals also struggle with successful recovery post-hospitalization, and increased challenges to receiving follow up care (Kay et al. 2014).

Similar to the challenges and needs of the homeless population, another complex and higher needs population with advanced medical problems is the geriatric population. The geriatric population is the fastest growing demographic in the United States, expected to almost double from 43.1 million in 2012, to 83.7 million Americans in 2050 (Ortman et al. 2014). One trauma center found that over the past decade, their incidence of geriatric trauma admission increased by 48%, with similar admission patterns found in other trauma centers (Lowe et al. 2018). As society continues to age, with elders more independent and active than the previous generations, the health care system must learn to adapt to this fast-growing demographic.

There are several factors that set the geriatric demographic apart from the general population yet make them similar to the homeless population. When recovering from traumatic injury, the cognitive and physiological decline associated with age can make recovery unpredictable (Engelhardt et al. 2018). Mental health issues are also a known risk factor when treating injury or illness among the elderly, with an estimated 15% of adults aged 60 and older suffering from a mental disorder (http://www.who.int/mediacentre/factsheets/fs381/en/ n.d.; Centers for Disease Control and Prevention and National Association of Chronic Disease Directors 2008). These patients are more likely to struggle with frailty and an elevated risk of illness and injury due to declining physiological systems (Engelhardt et al. 2018). This contributes to poorer outcomes, potentially leading to a loss of independence, increased social isolation and higher rates of re-admission to hospitals. Geriatric individuals may also present with atypical symptomology, which can complicate care management (Joseph et al. 2017). Specifically, with a traumatic injury, hospitalized geriatric patients are known to utilize more resources, suffer more medical complications and have an overall greater length of hospital stay (LOS) as compared to younger patients with similar injury severity. A multidisciplinary approach that offers psychiatric and gerontology consultations may lead to more cost-effective care and reduced LOS (McKevitt et al. 2003). Although there are published studies looking at geriatric, homeless individuals, there is little to no literature comparing two complex and vulnerable populations, geriatric and homeless, in a trauma setting (Hategan et al. 2016; Spiker et al. 2001; Brown et al. 2013; Brown et al. 2017; Brown et al. 2012).

Different trauma center levels have varying levels of resources to deliver trauma care. A Level I trauma center can provide total care for every injury aspect – from prevention through rehabilitation.. Level II centers are expected to be clinically equivalent to Level I centers in providing comprehensive definitive care, with the exception of complex specialized services such as replantation. The primary differences between Level I and II centers are minimum patient volume and trauma research publication requirements for Level I centers. Trauma activation occurs when the trauma center is notified that a trauma victim is coming so that a multidisciplinary trauma team consisting of surgeons, emergency physicians, nurses and other healthcare providers will be in the trauma resuscitation room prior to the patient’s arrival. Once the patient arrives, the trauma team performs continuous assessments and treatment to injuries until the patient is transferred to the operating room or intensive care unit (Committee on Trauma American College of Surgeons 2014).

Guidelines for field triage of injured patients to trauma centers have been promulgated by the Centers for Disease Control and promoted by the American College of Surgeons for adoption by trauma systems and trauma centers. Physiologic, anatomic, and mechanism of injury criteria form the basis for the majority of trauma patients’ triage; a short list of “special considerations” includes pregnancy, burns, young age, and older age. The triage process for trauma patients has criteria designed to identify those patients with a traumatic injury requiring a multidisciplinary rapid response of assessment, treatment, and management. The process is not perfect and is actively reviewed to ensure patients not requiring additional resources (over-triage) are not activated as a trauma response versus those patients who should be activated but are not identified in a timely manner and have a delay (under-triage). Geriatric patients are known as a population at risk for trauma activation under-triage (Chang et al. 2008). There are other cohorts of patients at risk for potential delayed or missed trauma triage response, but the growing geriatric population continues to demonstrate the need to reevaluate existing criteria to reduce under-triage.

Trauma centers have evolved and continually improved over the past 35 years to provide the best managed treatment and care to the most severely injured patients in the community. Trauma and emergent care are expensive and complex; thus, the over-use of resources in today’s healthcare industry is carefully reviewed. Frequent emergency department use and overcrowding are key issues in emergency management, and certain populations that are sicker or lack access to primary care contribute more to this issue than others (Bernstein 2006). This epidemiological review examines the elderly and homeless population treated at one Level II trauma center over a five-year period to identify any similarities and/or differences in medical care treatment and management of traumatic injury and outcomes between these two high-risk populations.

## Methods

A retrospective review of trauma registry data over 5 years (January 1, 2013 to December 31, 2017) at one American College of Surgeons (ACS) verified Level II trauma center in Southern California was completed, identifying all homeless (i.e., currently without a residential address at time of admission) and geriatric (age 65 and older) individuals. All patients were categorized in one of three patient groups – geriatric, homeless, and all other trauma patients. If a patient was elderly and homeless, the homeless cohort was chosen. Medical record and trauma registry data were collected and analyzed. Data elements collected included patient demographics, mechanism of injury, treatment, length of stay, comorbidities, complications, drug and alcohol screening results, and outcome. Descriptive analyses were completed. Data are presented as the mean ± standard deviation (SD) or the raw percentage score where appropriate. Population attributable risk was estimated to quantify risk in the exposed cohorts and calculated using the odds ratio as a point estimate of the Relative Risk (RR). Categorical data were analyzed using a two-sided Fisher exact test whereas Student t-test was used for continuous variables. Statistical analyses utilized R version 3.6.1, GraphPad QuickCalcs and MEDCALC statistical software. The *p* values were considered significant at *p* < 0.05.

## Results

Eight thousand four hundred thirty-one trauma patients were treated over the five-year period, and comprised of 3% (248) homeless, 30% (2545) geriatric and 67% (5638) all other trauma patients. Males were the predominant gender in all three groups, with the largest proportion within the homeless cohort (88% male). There were no readmissions of these patients, each visit represents one patient admission (Table 1).

**Table 1:** Patient and Event Characteristics

	Homeless	Geriatric	All Other Patients
	N	N	N
**Number of patients**	248 (3%)	2545 (30%)	5638 (67%)
**Age**			
*< 65 years*	230		5638
*65 years and older*	18	2545	
**Gender**			
*Female*	30 (12%)	1249 (49%)	1536 (27%)
*Male*	218 (88%)	1296 (51%)	4102 (73%)
**Hospital Length of Stay (days)**			
*Mean ± SD*	11.7 ± 21.4	5.5 ± 7.5	4.2 ± 7.2
*Median*	4	3	2
*Range*	1 to 145	1 to 136	1 to 143
**ISS**			
*Mean ± SD*	11.3 ± 11.0	10.5 ± 7.7	9.5 ± 9.0
*Median*	9	9	6
*Range*	1 to 75	1 to 75	1 to 75
**Non-Trauma Activated Patients**	23%	41%	16%
**ICU Admit**	113 (46%)	1052 (41%)	1648 (29%)
**Positive BAC screen**	122/232 (53%)	201/1884 (11%)	1523/5021 (30%)
**Positive Drug Screen**	72/110 (66%)	17/214 (8%)	442/1480 (30%)
**Deaths**	12 (5%)	175 (7%)	152 (3%)

Homeless trauma patients are 1.2 times more likely to be male as compared to non-homeless trauma patients (RR 1.20, *p* < 0.0001). This gender disparity evident among the homeless is consistent with what is seen statewide in California and nationally, where estimates of male homelessness has been reported to be approximately 70% (The U.S. Department of Housing and Urban Development n.d.; Regional Task Force on the Homeless n.d.). The largest proportion of female trauma patients (49%) were geriatric trauma patients. They are almost two times more likely to be female as compared to all other trauma patients (RR 1.84, *p* < 0.0001). Average age for homeless was 48 years, 80 years for geriatric, and 37 years for all other patients.

**Fig. 1:**
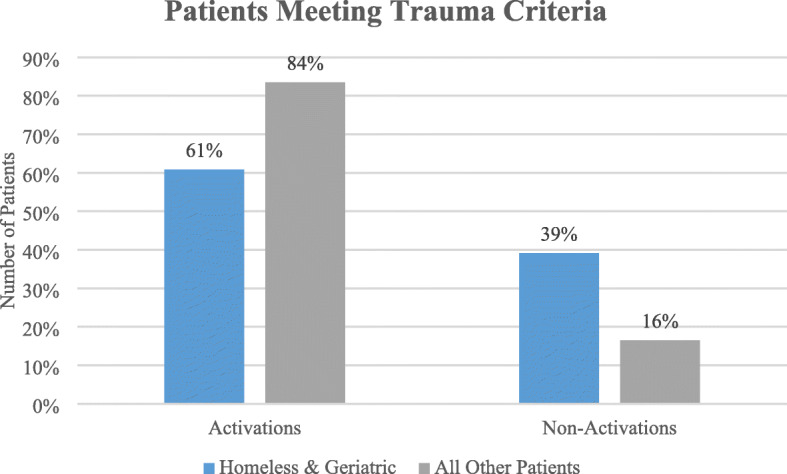
Trauma activation by patient cohorts

Trauma activation criteria was met for 84% of all other trauma patients, yet only 61% of homeless and geriatric patients combined (see Fig. 1). Trauma surgeons were consulted for patients not meeting trauma criteria after being seen in the Emergency Department and later determined to need a higher level of care and upgraded to the trauma service (initially under-triaged patients). Homeless patients were found to be 1.4 times more likely to be admitted to the trauma service by non-activation (RR 1.44, *p* = 0.0026) but geriatric patients were approximately three times more likely to be a non-trauma activation trauma admission (RR 2.56, *p* < 0.0001) as compared to all other trauma patients. Thus, under-triaged patients were more often homeless and geriatric (39%) as compared to only 16% of all other trauma patients.

Both homeless (46%) and geriatric (41%) cohorts were more often admitted to the ICU as compared to all other trauma inpatients (29%), which reflects higher acuity, mortality rates and increased length of stay (LOS) in both cohorts compared to all other trauma patients. Homeless and geriatric trauma patients are 1.6 times (RR 1.56, *p* < 0.0001) and 1.4 times (RR 1.4, *p* < 0.0001) more likely to be admitted to the ICU, respectively. Both cohorts had more complicated care management due to comorbidities, often due to alcohol and drug use and, in the homeless cohort, pre-existing psychiatric history. Geriatric patients more often arrived with multiple comorbidities and history of chronic disease (64%) which contributed to a complex hospital stay and challenging discharge planning (Table 2).

**Table 2:** Comorbidities by Patient Group

Comorbidities	Homeless	Geriatric	All Other Patients	Total
*Obesity*	6 (2%)	91 (4%)	256 (8%)	353 (6%)
*Alcohol Dependence*	113 (29%)	127 (6%)	469 (14%)	709 (12%)
*Drug Dependence*	55 (14%)	14 (1%)	539 (16%)	608 (11%)
*Psych History*	53 (14%)	386 (19%)	668 (20%)	1107 (19%)
*Seizures*	34 (9%)	72 (3%)	154 (5%)	260 (5%)
*Smoker*	87 (22%)	60 (3%)	654 (20%)	801 (14%)
*Disease*	44 (11%)	1310 (64%)	543 (17%)	1897 (33%)

The predominant mechanism of injury for both homeless and geriatric cohorts was fall from same level. Mechanism of injury for the homeless cohort was diverse yet reflective of their environmental exposures: Falls (38%), pedestrian/bicycle related (27%) and assault related (24%). Homeless individuals injured by pedestrian and bicycle versus auto mechanism had the most severe injuries, the most complex treatments and procedures, and the highest mortality rate. The most common mechanisms of injury for the geriatric group were falls (76%), motor vehicle related injuries (12%) and pedestrian/bicycle-related injuries (6%), without the additional comorbidity of presence of alcohol and/or drugs as seen among the homeless.

**Fig. 2:**
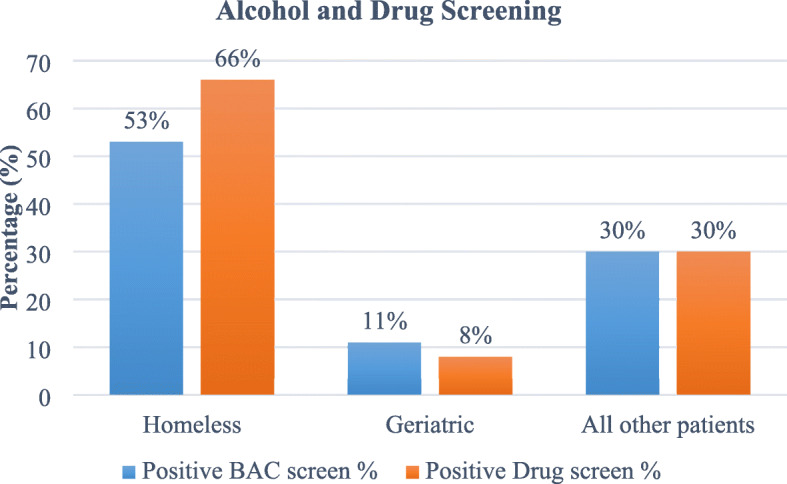
Positive toxicology screen by patient cohorts

Toxicology and blood alcohol content (BAC) screens are not routinely completed for all trauma patients. Of the population tested in this study, homeless patients were more often under the influence of drugs and/or alcohol at time of injury. Fifty-three percent tested positive for blood alcohol content (BAC) and 66% positive for illegal drugs. Positive BAC among the homeless cohort was higher as compared to the geriatric (11% positive BAC) and all other trauma patients (30% positive BAC). Homeless trauma patients are three times more likely to arrive with positive BAC as compared to all other trauma patients (RR 3.05, *p* < 0.0001) and approximately four times more likely to have a positive toxicology screen on arrival to the hospital (RR 3.70, *p* < 0.0001). In comparison, geriatric trauma patients who were screened are less likely to have a positive drug screen (RR 0.09, *p* < 0.0001) as compared to all other trauma patients. However, drug testing for presence of illegal drugs on admission was not routinely done, with 92% of geriatric and 74% of all other trauma patients not being tested but all were screened clinically by a social worker. The homeless cohort had the highest number of drug screens completed (44%) and, of those patients, 66% screened positive. (See Fig. 2) The actual number of homeless patients testing positive for illegal drugs on admission is most likely much higher. Toxicology screening is now more routine for all trauma patients in our trauma center and future review will continue to monitor these results.

There were a small group of trauma patients identified as both geriatric and homeless (18). For all other analyses, those eighteen patients were grouped in the homeless cohort. This sub-cohort was predominantly male (89%) with average hospital stay of 8 days and with a fall mechanism of injury (50%), reflective of the most common mechanism for the elderly. The remaining included assault (17%), pedestrian struck by autos (17%) and being struck by auto while riding a bicycle (17%). Nearly 78% were trauma activations, with 22% under-triaged. Although 50% had a positive BAC, only 1.5% had a positive drug screen.

Injury severity was not significantly different between the three groups of geriatric, homeless and all other trauma patients, but there was a higher rate of death within both geriatric (7%) and homeless cohorts (5%) as compared to all trauma patients (3%) (Table 3). Both homeless (RR 1.79, *p* = 0.0457) and geriatric (RR 2.4, *p* < 0.0001) trauma patients are approximately two times more likely to die during hospital stay as compared to all other trauma patients. In addition, the increased number of complications associated with their injuries, extensive comorbidities, and increased length of hospital stay all reflect the complexity of care involved, especially with the homeless. This complexity of care and duration of hospital stay are reflected in the hospital charges. The average hospital charge for a homeless trauma patient is higher than for geriatric trauma patients ($252,377 vs $144,331) but similar to the average charge for all other trauma patients ($263,373).

**Table 3:** Injury and Outcomes by Homeless, Geriatric and All Other Patients

	Homeless	Geriatric	All Other Patients
**Number of patients**	248 (3%)	2545 (30%)	5638 (67%)
**Major Trauma ISS > 16**	58 (23%)	533 (21%)	952 (17%)
Mean ISS ± SD	26.8 ± 12.0	22.0 ± 7.0	25.0 ± 10.7
**Major Surgery Required**	77 (31%)	510 (20%)	1467 (26%)
**Deaths**	12 (5%)	175 (7%)	152 (3%)
**Hospital Length of Stay (days)**			
*Mean ± SD*	11.7 ± 21.4	5.5 ± 7.5	4.2 ± 7.2
*Median*	4	3	2
*Range*	1 to 145	1 to 136	1 to 143
**Cost of Stay**			
*Mean ± SD*	$252,376.53 ± $431,170.54	$144,330.58 ± $224,236.79	$263,372.36 ± $9,350,546.45
*Median*	$431,170.54	$73,230.26	$53,886.92
*Range*	$9506.42 to $3,448,886.15	$2163.00 to $2,929,948.43	$1082.00 to $701,810,283.00
**Mean BAC (mg/dl)**	133 ± 156	18 ± 62	61 ± 111

## Discussion

There is a growing interest and need for trauma centers to offer specialized care for elderly patients. Geriatric patients need their own guidelines and treatment plans in all phases of healthcare, including trauma and critical care. The same is needed for patients with socioeconomic hardship, psychiatric history and drug and alcohol issues. Each of these comorbidities can be found within any cohort at a trauma center, but the homeless often have it all, compounded by factors such as lack of a support system or place to stay after discharge. Their admission and care can be very difficult for a hospital to manage. Psychiatric active symptoms and drug use clearly afflict a high percentage of our homeless population and most likely the driver behind their homelessness status. Whether these symptoms or drug use is active or not tends to affect follow up care or placement. This results in injured patients presenting with a multitude of comorbidities that add complexity to their treatment.

Furthermore, homelessness itself presents an increased risk factor for death, independent of socio-economic standing and morbidity (Morrison 2009). Homeless trauma patients comprise a smaller cohort of patients who are treated in the community, but the homeless patient’s hospital admission and discharge planning complexities encompass many similarities to the geriatric patient. Both groups require additional time by social workers and case managers for their complex case management and discharge planning, even more so for the geriatric homeless patient. Recovery in a home with the support of family or friends is not an option for most; thus, rehabilitative and board and care facilities are often the best option. Unfortunately, the availability of beds in these facilities is very limited, leading to extended hospital stays and charges. Once discharged, future contact is very difficult, often impossible. Thus, the likelihood of the patient returning for follow-up care is minimal to none. As a result, repeat visits to the ED and/or trauma service with subsequent readmissions are frequent, especially more common for the homeless patient where the risks for infection and injury are substantially higher in that environment.

Especially for those living with traumatic brain injuries, barriers associated with finding appropriate living environments for these individuals include lack of brain injury-specific facilities, beds and trained staff, poor coordination of services, and long waiting lists for specialized residential settings (Colantonio et al. 2010). In addition to the need for individualized treatment after discharge, brain injured individuals who are also homeless may make recovery from traumatic injury even more difficult due to poor access to care following discharge (Colantonio et al. 2010; Laliberté et al. 2020). Compared to housed patients, people experiencing homelessness are less likely to make primary care visits and have high emergency department utilization (Hwang and Burns 2014). Laliberté and colleagues found that homeless adults with mental illness at discharge were significantly more likely to have a readmission within 30 days following discharge and to have an ED visit. Individuals discharged as homeless were less likely to visit the doctor’s office within 30 days following discharge, while more likely to have used acute care services. This suggests that homelessness is associated with poor access to care following discharge despite the higher need for care continuity. Thus, it is imperative to improve access to health services to reduce acute care service use and improve care continuity (Laliberté et al. 2020).

Our findings are consistent with previous studies. A quasi-systematic review that identified several barriers or facilitators to obtaining primary care that were associated with being homeless. Barriers include being male, having comorbid conditions like mental illness, faced with competing priorities (e.g. difficulty finding food, shelter, clothing, or a place to wash), and having the lack of health insurance. Facilitators ranged from tailored health care delivery systems to having a regular source of care (White and Newman 2015). Other studies have found that homeless individuals have low use of medical services relative to their needs and are not getting adequate healthcare services even when their health places them at high risk of death. (Hwang et al. 2001; Stein et al. 2007; O’Toole et al. 1999; Padgett et al. 1990) Another study looking at homeless veterans found that although mental illness did not pose a specific barrier to initiating medical care, specific diagnosis such as substance use or schizophrenia were related to a lower likelihood of receiving three or more medical visits (Desai et al. 2003). A major institutional barrier health service accessibility by homeless people is the lack of systematic coordination at different services locations, all with separate admission procedures (Drury 2003). A demonstration clinic that integrated homeless, primary care, and mental health services for homeless veterans was found improve access to primary care services and reduced emergency services (McGuire et al. 2009).

Our review focused on the most injured patients, requiring a higher level of emergent care. But the challenges faced by the emergency physician treating these two groups of patients are more frequent and multifaceted. For the elderly, obtaining relevant historical information in the setting of age-related cognitive decline is difficult at best. Often, additional efforts must be made to reach out to family, friends or care facility staff to obtain appropriate information relating to the presenting complaint. This impacts the initiation and direction of care. Once initiated, the biggest challenge is varied physiologic expression of disease in the elderly, especially as it is manifested in pain response. Parameters such as heart rate, blood pressure and vocal expressions of discomfort used in the younger age population are much less reliable in the elderly. Once a diagnosis is established, the appropriate disposition is complicated by issues surrounding the patients coping abilities. Complicated home environments (e.g., stairs, loose rugs, etc.), lack of social support network, and weakened ability to understand discharge instructions preclude the creation of an effective discharge plan. The homeless population shares many of the same challenges for the ED practitioner as those seen in the elderly. Mental health and substance abuse issues frequently complicate communication regarding presenting complaints. Additionally, many have smoldering underlying chronic disease that has yet to be diagnosed but greatly impacts presenting physiology, making treatment especially challenging. To create an effective disposition plan, one must address the same complex multitude of social issues we face with the elderly. In addition, frequently it is necessary to assume the homeless patient will not seek appropriate follow-up due to issues surrounding access or desire. Consequently, more aggressive strategies for treatment are often necessary up front to mitigate deterioration in the patient’s condition.

It is important to address the practical challenges involved in dealing with homelessness, injury and disease, and assess structural and environmental amendments that can improve quality of life. Inpatient and discharge planning management of the hospitalized homeless may affect hospitals differently, depending on the homeless population within the community it serves, but it is evident that providing dedicated social work and case management services are critical (Morris and Gordon 2006). In 2018, the State of California enacted a law that sets guidelines on and requires comprehensive discharge planning for the homeless hospital patient. It requires the facility to offer referrals for board and care facilities and mental health care and must keep track of where each homeless patient has been discharged (https://leginfo.legislature.ca.gov/faces/billTextClient.xhtml?bill_id=201720180SB1152 n.d.). While our trauma center already has dedicated social workers who implement these policies, this bill can potentially reduce a lot of the inherent risks of a recovering patient discharged onto the street, as housing and case management has been shown to reduce hospital stays and emergent visits, also lessening the burden on the health care delivery system (Sadowski et al. 2009).

There is growing interest in research that has aimed to identify the issues within these vulnerable populations and improve quality of care. Several studies have reviewed the impact of geriatric frailty after traumatic injury. The Trauma Specific Frailty Index is a measure used to determine frailty and predict outcomes (Joseph et al. 2017; Joseph et al. 2014). Developing a geriatric specific hospital protocol alone either was not found to create a significant reduction in mortality, or had mixed results (Saillant et al. 2017; Bradburn et al. 2018). However, studies have found that providing frail geriatric patients with specialized care, including Social Work Intervention, Hospitalist consult, and family engagement in discharge planning, decreased LOS, loss of independence, and 30-day readmission rates (Engelhardt et al. 2018; Morrison 2009; Joseph et al. 2014). A multidisciplinary approach, including a geriatrics consultation, is essential for patients with multiple morbidities (de Vos et al. 2016; Fallon Jr et al. 2006). It is important to acknowledge the complex interplay of various factors contributing to emergent medical issues in the geriatric population, and to take a holistic view of treatment (Duckworth 2018). In the past, our trauma center did not routinely use frailty screening, but after a review of the research and the population in questions, a frailty protocol is being implemented.

Current trauma triage activation guidelines may need revisions to account for specialized patients who fall out of activation criteria, the under-triaged patient. These are the patients not meeting trauma activation criteria set by the county but, upon arrival, a closer look by a trauma surgeon determined the patient required a higher level of care. There is an unconscious age bias that effects health care workers both in and out of the field, with geriatric patients less likely to be referred to a trauma center, as compared to younger patients with similar injuries (Chang et al. 2008; Lane et al. 2003). The under-triage rate of elderly trauma patients has been found to be as high as 49.9% (Chang et al. 2008). The time between patient arrivals to trauma upgrade is currently being closely monitored in our trauma center and evaluated for efficiency and opportunities for improvement, including a thorough review of each under-triaged patient. The goal is a process that benefits all patients equally to ensure that the best medical care and management are provided. Our trauma triage model was updated shortly after our cohorts were identified for study. A review of data after these changes were implemented have shown some differences in triage outcomes for all trauma patients including an overall decline in major activations, an increase in minor activations and increase in trauma consultation among homeless patients arriving in the Emergency Department. The use of observation units prior to formal admission has increased for all patients, especially among the elderly.

## Conclusion

The multidisciplinary approach to trauma care is necessary and has worked successfully toward the goal of saving lives and improving outcomes for the most critically injured, but it is not perfect. It is prudent to continually review the population you are serving and identify those patients requiring additional resources and have increased length of stay or difficulty with discharge planning. The geriatric and homeless populations are known to have challenges with healthcare and a need of tailored medical management but, when acute traumatic injuries are added, these two groups face even more challenges to receive the medical treatment and critical management best for their survival and recovery. These findings further emphasize the need for policies to provide more resources to a public health or health care workforce to manage the necessary needs of the homeless and geriatric populations. Through partnerships with local and state agencies, an integrated, multidisciplinary health care team with an outreach focus can be formed to provide continuity of care, ensure quality of care, and help those vulnerable become self-sufficient. It is important to create an evidence-based approach for services and care in order to achieve the best possible patient outcomes, and to lessen the strain on the social safety net of emergent medical treatment. Overburdening trauma centers could result in mismanaged patients discharged too soon without receiving all the services they need and experience a lack of follow up care. This, ultimately, will add to healthcare inequity if not carefully and proactively monitored.

## Data Availability

Datasets will not be shared due to institutional restriction in data use agreement but data supporting the findings of this study are available from the authors upon reasonable request.

## Abbreviations

ACS: American College of Surgeons
AVG: Average value in set of data
BAC: Blood Alcohol Concentration
ED: Emergency Department
ICU: Intensive care unit
ISS: Injury severity score standardization of severity of traumatic injury
LOS: Length of stay in day units
SD: Standard deviation
TBI: Traumatic Brain Injury
